# Examining the Cognitive, Practical, and Emotional Demands of Managing Physical Activity in Adolescents With Type 1 Diabetes: A Qualitative Study With Adolescents, Parents, and Healthcare Professionals

**DOI:** 10.1155/pedi/4578096

**Published:** 2025-12-19

**Authors:** Emma Joanne Cockcroft, Jane Rebecca Smith, Jenny Lloyd, Louie Johnson, Richard Pulsford, Ross Clarke, Parth Narendran, Gina Gardener, Tallulah Ngamy, Renuka Priyani Dias, Robert Charles Andrews

**Affiliations:** ^1^ Department of Health and Community Sciences, University of Exeter, Exeter, UK, exeter.ac.uk; ^2^ Institute of Immunology and Immunotherapy, University of Birmingham, Birmingham, UK, birmingham.ac.uk; ^3^ Department of Paediatric Endocrinology, Birmingham and Women’s NHS Foundation Trust, Birmingham, UK; ^4^ Institute of Applied Health Science, University of Birmingham, Birmingham, UK, birmingham.ac.uk; ^5^ NIHR Exeter Biomedical Research Centre, University of Exeter, Exeter, UK, exeter.ac.uk; ^6^ Department of Diabetes, Taunton and Somerset NHS Foundation Trust, Taunton, UK

**Keywords:** exercise, support strategies, type 1 diabetes, young people

## Abstract

**Objective:**

Adolescents with type 1 diabetes (T1D) face unique barriers to physical activity (PA), and most do not meet recommended targets despite its recognised health benefits. To address the lack of tailored, evidence‐based support for this group, this study explores how adolescents manage PA and how it is influenced by the wider support system, including parents, carers, and healthcare professionals (HCPs).

**Research Design and Methods:**

Semi‐structured interviews were conducted with adolescents with T1D (*n* = 11), parents/carers (*n* = 15), and HCPs (*n* = 11). Adolescents were aged between 12 and 18 (64% female). HCPs were dieticians (*n* = 7), nurses (*n* = 2), a doctor (*n* = 1) and a health and wellbeing practitioner (*n* = 1). Interviews explored practical, emotional, and contextual factors influencing PA. Data were analysed using thematic analysis.

**Results:**

Participants described cognitive, emotional, and practical demands of managing T1D during PA. Thematic analysis identified three overarching themes: (1) the mental effort required to manage diabetes with PA, including parental anxiety, desire for normality, and unpredictability of glucose responses; (2) practical and organisational challenges, such as access to supportive environments, technology, and activity‐specific logistics; and (3) adaptive management strategies, including trial and error, parental involvement, peer learning, and variable clinical support. Current support was often generic, leading families to rely on self‐devised strategies and informal networks to support their individual needs.

**Conclusion:**

Enhanced, youth‐friendly, and activity‐specific guidance is needed for adolescents with T1D. This should include training for healthcare professionals, teachers, and coaches. Future work should prioritise the co‐design of resources and interventions with young people and families, integrating structured peer support.

## 1. Introduction

Type 1 diabetes (T1D) is a common chronic autoimmune condition affecting over 1.5 million children and adolescents worldwide with prevalence rising annually [1]. Despite well‐established physical activity (PA) guidelines recommending at least 60 min of daily moderate‐to‐vigorous activity for young people [2], ~70% of children and adolescents with T1D fail to meet these targets [3]. Compared to their peers without diabetes, young people with T1D have lower levels of PA, higher levels of sedentary behaviour, reduced participation in organised sports, and lower cardiorespiratory fitness [4]. This lower level of PA is especially concerning given that adolescents with T1D have twice the lifetime risk of developing cardiovascular disease (CVD), a leading cause of morbidity and mortality, compared to those without T1D [5].

Regular PA is an important component in the management of T1D and is associated with a number of health benefits, including improved metabolic control, body composition, quality of life, and mental wellbeing [6, 7]. Despite potential benefits, adolescents with T1D face unique challenges in achieving recommended target levels of PA. These include fear of hypoglycaemia around exercise, the variable physiological effects of PA on blood glucose, and practical barriers related to diabetes management such as insulin adjustments, carbohydrate intake, and the burden of monitoring [8].

While these barriers to PA have been previously reported, there remain significant gaps in our understanding of how adolescents with type 1 diabetes, their parents or carers, and healthcare professionals (HCPs) actively manage their diabetes around PA. Most existing qualitative studies focus on older adolescents or adults. This leaves younger adolescents, who are at a critical transitional phase of increasing self‐management responsibility [9], and at a time where PA levels have been shown to decline [10, 11], underserved. Early intervention during adolescents is important to preserve activity levels for better health outcomes in later life [12]. Furthermore, current interventions aimed at increasing PA in youth with T1Dare sparse [13–15], often lack theoretical grounding, rarely involve co‐design with patients and families, and tend to focus on structured exercise without addressing the broader psychosocial and logistical challenges of PA participation. There is limited tailored support beyond clinician facing International Society for Pediatric and Adolescent Diabetes (ISPAD) guidance [16] and no evidence‐based patient centred interventions that have been tested in randomised controlled trials or designed using robust approaches to intervention development.

This gap in understanding and provision of tailored PA support is important because achieving effective, sustainable increases in PA requires support strategies that are informed by the lived realities and preferences of young people with T1D and those involved in their care. Without tailored approaches, interventions risk poor uptake and limited impact. Recent surveys within the UK highlight HCPs’ limited formal training and lack of practical resources to support PA integration in adolescents with type 1 diabetes, exacerbating this unmet need [9].

This study aims to address this unmet need by exploring multi‐stakeholder perspectives, including adolescents with type 1 diabetes, their parents/carers, and HCPs, to understand the practical, emotional, and contextual factors influencing participation in PA. Through qualitative methods, we seek to capture the strategies currently used to overcome challenges, identify unmet support needs, and inform the co‐development of tailored interventions that reflect the complexities of managing T1D during adolescence.

## 2. Methods

### 2.1. Study Design

This qualitative study employed semi‐structured interviews conducted within a constructivist epistemological framework, which recognises that individuals construct meaning through their lived experiences of managing paediatric T1D and PA. This approach is particularly well‐suited to understanding the contextually shaped experiences and perspectives of adolescents, parents, and healthcare professionals navigating PA management in T1D [17]. The study adhered to the Consolidated Criteria for Reporting Qualitative Research (COREQ) checklist to ensure transparency and rigor in reporting [18]. (see Supporting Information 1: Appendix 1). Further details on study design, conduct, and data analysis methods are provided below.

This study was conducted in accordance with the Declaration of Helsinki and received ethics approval from the University of Exeter Research Ethics Committee prior to participant recruitment.

### 2.2. Participants and Recruitment

Information about this study was disseminated to potential participants through several channels. Healthcare professionals were invited via the Children and Young People’s Diabetes Network (https://www.cypdiabetesnetwork.nhs.uk). Adolescents with T1D and their parents/carers were recruited through study advertisements circulated by Diabetes UK and Breakthrough T1D on their official communication channels, including websites, newsletters, and social media. Additional social media outreach included posts on relevant Facebook groups, Twitter/X and Instagram using diabetes‐related hashtags. All individuals who expressed interest in participating were sent patient information sheets and further instructions on how to contact the research team if they wished to join the study. The research team followed up each contact to organise interviews, sending reminders 1 week later if no response was received.

All participants received age‐appropriate written information sheets prior to participation. For adolescents aged under 16 years written informed consent was obtained from parents/guardians, and written assent was obtained directly from the adolescents themselves. This dual consent process was followed for all adolescent participants, including those whose parents did not themselves participate in the study. For parents/carers, adolescents over 16 years and healthcare professionals, written informed consent was obtained directly from the participant. Informed consent was documented in writing prior to each interview, using one of the following methods: (1) completion of an electronic consent form; (2) consent via email, with participants indicating agreement to each consent statement by reply email and signing with name and date; or (3) verbal consent documented via a transcript of the consent interaction.

### 2.3. Data Collection

Semi‐structured interviews were conducted over video call using either Teams or Zoom software. Interviews were conducted between October 2023 and April 2024 by EC (a female postdoctoral researcher with 9 years’ experience of mixed methods research, with a focus on PA and T1D). Interviews were conducted using a semi‐structured topic guide which was developed in partnership with the project Young Persons Advisory group (see Supporting Information 2: Appendix 2). The topic guide was designed to explore both barriers and enablers to PA, with several interview questions specifically inviting participants to reflect on difficulties, negative experiences, and challenges. Participants were not known to the research team prior to interview.

Recruitment continued until subsequent interviews produced minimal or no new insights relevant to the study aims, and the research team was confident that further data would not alter the thematic map. Practical constraints, including slow participant recruitment and time limitations imposed by the study schedule, also informed the final sample size. This pragmatic and iterative approach aligns with contemporary guidance on assessing data adequacy and sample sufficiency in qualitative research [19].

General demographic data and brief assessment of PA levels using the single item PA recall [20] was used as a brief screen for PA levels of adolescent participants and the adolescents of the parents/carers, to help us understand and describe the demographics of the participant group.

### 2.4. Data Analysis

The audio‐recorded interviews were transcribed verbatim by a professional transcriber before being imported into NVivo (V14.0) for data organisation and to facilitate analysis. Analysis was primarily inductive and aimed to explore barriers, facilitators and contextual factors influencing PA participation, as well as the acceptability and suitability of support strategies among adolescents with type 1 diabetes, and those supporting them. Analysis was informed by a thematic approach [21] consisting of 5 stages; Stage 1: Familiarisation: Two researchers (GG and EC) familiarised themselves with the data by reading three different transcripts (i.e., six per group in total) from each participant group (adolescents, parents and HCPs), and making analytical notes on content relevant to the study aims; Stage 2: Initial coding: Two researchers (GG and EC) independently coded three transcripts per group using descriptive, inductive codes. stage 3: reviewing codes across transcripts and grouping into initial broader themes. This stage was carried out by 4 researchers, including a young person with T1D (EC, GG, TN and RC). Stage 4: Development of coding framework to apply across transcripts. This was then applied by three researchers double coding all transcripts (EC, GG and TN). Stage 5: Discussion of codes and themes with the wider research team to develop the final set of codes and themes presented in this paper. To maintain rigor and transparency, researcher reflexivity was actively integrated throughout the study by regularly engaging in critical self‐reflection regarding potential biases, blind spots, and the framing of questions posed to the data.

## 3. Results

### 3.1. Sample Characteristics

Thirty‐seven participants took part in semi‐structured interviews: 11 adolescents (7 female, 4 male; median age 12 years, range 12–18), 15 parents/carers (14 female, 1 male; median age 45 years, range 35–55), and 11 healthcare professionals (10 female, 1 male; median 7.5 years’ experience in paediatric diabetes, range 3–21 years). Of the 19 eligible adolescents who expressed interest in the study, all were contacted and 11 took part in interviews. Similarly, of 22 eligible parents/carers and 18 healthcare professionals, all were contacted and 15 and 11, respectively, were interviewed. Those not enrolled most commonly withdrew due to scheduling challenges or did not respond following contact. Nine of the adolescents interviewed also had a parent as part of the sample. Sample characteristics are summarised in Table 1.

**Table 1:** Sample characteristics.

Characteristic	Adolescents (*n* = 11)	Parents (*n* = 15)	HCPs (*n* = 11)
Sex (male/female), *n*	4/7	1/14	1/10
Median age (range)	12 (12–18)	45 (35–55)	34 (25–46)
Mean type 1 diabetes duration of adolescent, median (range)	3 (1–11.5)	4 (1–13)	—
Years clinical experience (HCPs)	—	—	7.5 (3–12)
Insulin regimen, n(MDI/pump/closed loop)^a^	3/3/5	3/7/5	—
Age of child, years (SD)^a^	—	14.5 (1.4)	—
Child sex (male/female), *n* ^a^	—	9/6	—
Ethnicity	White or Caucasian *n* = 10 Mixed Black/White *n* = 1	White or Caucasian *n* = 14 Hispanic *n* = 1	White or Caucasian *n* = 10 Mixed *n* = 1

^a^Parent‐reported details of their child with type 1 diabetes.

PA levels, as assessed by the screening questionnaire, showed that four adolescents engaged in at least 60 min of moderate‐to‐vigorous PA (MVPA) every day in the previous week. Two adolescents reported being active for 6 days, four for 5 days, and one for 3 days within the same period. This equates to ~36% of participants meeting PA recommendations of 60 min MVPA per day. This is in line with previously reported PA in this population which found that between 35% and 39% of adolescents with T1D and 57%–60% of the controls met the recommended 60‐min duration of MVPA [3] Parent‐reported data indicated that six children achieved the recommended 60 min of MVPA on all 7 days, with two being active on 6 days, three on 5 days, one on 4 days, one on 3 days, and two on 2 days during the previous week.

Interviews ranged in length from 33 to 55 min for HCPs, 26 to 64 min for parents, and 25 to 43 min for adolescents.

### 3.2. Thematic Analysis: Experiences of Physical Activity and Type 1 Diabetes

Analysis of the interview data led to the development of three central themes; (1) How much mental effort it takes to manage T1D when being physically active; (2) Practical and organisational challenges of doing PA with T1D; and (3) Ways people manage their T1D when doing PA. All themes and their sub‐themes were identified across adolescents, parents/carers, and healthcare professionals, with representative quotes from each group provided in Tables 2–4.

**Table 2:** Theme 1: How much mental effort it takes to manage type 1 diabetes when being physically active.

Sub‐themes	Example interview quotes
Parental anxiety: Parents experience ongoing concern for their child’s safety during physical activity, leading to close monitoring and sometimes restricting independence.	*“I still feel I have … I want to be there and just make … just make sure and it’s 2 h he’s away for*, *umm…at training and*, *of course*, *I’m constantly watching him and constantly watching my phone to check his levels*, *umm … so*, *reasonably confident but not … not to the point of I can relax.”* (*Parent 9*)
	*“I think barriers in the younger ones is parental anxiety”* (*HCP 5*)
	*Mmm*, *mmm. It’s not really for me*, *it’s for my mum and dad ‘*cos *they are … they’re*, *like*, *they’re always watching it … and they’re always worrying* (*Adolescent 1*)
Not wanting to feel different: Adolescents and parents describe efforts to avoid stigma or standing out due to diabetes management during sport or group activities.	*“…yeah*, *I mean*, *I think the only worry is*, *perhaps*, *maybe*, *umm*., *not as vocal about*, *maybe*, *yeah … I still feel that*, *maybe*, *he would stay on the pitch*, *to not let down teammates…rather than come off and treat a low. He’d still feel*, *probably*, *guilty at doing that.”* (*Parent 5*)
	*“…but and*, *also … I…I can’t*, *like*, *like*, *umm … like*, *everything can’t revolve around me and what might*, *what then…’*cos *of my diabetes so it’s just*, *that’s annoying”* (*Adolescent 5*)
	*“I guess the final barrier*, *I’d say*, *is … is the tech so people are worried that*, *you know*, *if they’re in sport clothes*, *like a swimming costume*, *where their sensor’s gonna be out*, *then that might be another barrier”* (*HCP 11*)
Variability: Blood glucose responses vary according to activity and context, creating unpredictability and frustration for families and young people.	*“it’s just so unpredictable and that’s frustrating … sometimes it … it just … can’t think of an example*, *really*, *but sometimes you’re*, *like*, *it doesn’t … ah … these things are all very well in theory but every day can be different*, *you know……and I think … the hardest thing is I like to be in control. I like to make sense of things and then be able to make good decisions and*, *you know*, *it was so frustrating to … have different results all the time*, *you know…”* (*parent 12*)
	*“Umm*, *I think the main thing is just all different sports affect your levels differently.”* (*Adolescent 7*)
	*” I guess one thing would be … for the adolescents*, *in particular*, *the difference between exercising in a gym and pumping weights… “*(*HCP2*)
Decision making for physical activity and negatives of physical activity with type 1 diabetes: Managing type 1 diabetes in activity requires frequent, complex decisions, which sometimes result in missed opportunities to participle or reduced enjoyment.	*“when I’m*, *like*, *hypo I have to sit out for*, *like*, *15 minutes or when I’m high I don’t play as well because when I’m like in range and perfect*, *I’m … good and I’m just in a better mindset.”* (*Adolescent 3*)
	*“it’s another thing to … to manage … it … umm … and … so I think*, *yeah*, *there’s definitely a*, *kind of*, *a reticence for … the ones who like it and they’re doing it anyway they … they’re doing it anyway and some of them … are doing it anyway even though it’s causing all sorts of problems. I*, *actually*, *‘we could help with this…’ umm … is it … but I think the ones who*, *maybe*, *find that a bit scary or … or … or don’t want to do it*, *they can be quite difficult to*, *umm … encourage to do even a little and even just*, *you know”* (*HCP 09*)
	*“Sometimes she has to eat dinner not long before she goes in and the insulin can really just kick in and she starts to be active and plummet her down and she can spend*, *you know*, *forty-5 minutes sitting out waiting for her bloods to recover”* (*Parent 12*)

**Table 3:** Theme 2: Practical and organisational challenges of doing physical activity with type 1 diabetes.

Sub‐themes	Example interview quotes
Access: Limited resources, technology, or inclusive opportunities can restrict participation in PA for young people with T1D.	*“I’m a goalie so I have my … my glove bags at the side with all my*, *like*, *with sugar in it and with my … my pod and my phone … not my pod*, *the … the handset*, *like … device for my pump which’ll … I’ve got that at the side so that helps me a lot*, *just … I’ll just*, *every once in a while I’ll go over and I’ll check”* (*Adolescent 10*)
	*“I have had one football coach recently say to me he won’t play him because he’s worried about him”* (*Parent 2*)
	*“It’s basically a signposting exercise and they’ve got different people offering different things that kids can go and sign up for*, *but they have to make the effort to go and do it. You can’t refer in*, *they don’t let you go if you’ve got*, *kind of*, *any other health complications”* (*HCP 3*)
Logistical barriers to PA relating to type 1 diabetes: Carrying supplies and dealing with unpredictable activity schedules make diabetes management during PA harder.	*“Sometimes*, *umm … the pitches would be enormous or wouldn’t have*, *like*, *quite close access to him*, *sometimes he’d be asked to put his bags and belongings far away from where he was playing sports and … that would be a nightmare too. Sometimes he would dump it in the pouring rain and you would know there’d be*, *like*, *bits of kit*, *like*, *getting soaked and full of mud.”* (*Parent 5*)
	*like*, *sometimes when I’m hypo I don’t realise it and I’m too far away from my phone for the alarms to go off on my Libre…*(*Adolescent 3*)
	*well*, *I know I need to reduce insulin before they do PE but sometimes they go to PE class and they don’t do anything so I’ve reduced their insulin and then they’re running high.’ Yeah*, *I see that or ‘they go to PE class and then it’s cross country and we haven’t reduced it enough’*, *so…*(*HCP 8*)

**Table 4:** Theme: Ways people manage their type 1 diabetes when doing physical activity.

Sub‐themes	Example interview quotes
Trial and error: Finding what works for PA with T1D is a process of ongoing experimentation.	*“It is trial and error*, *though*, *and it is … the problem is it’s not an exact science and … there is no … right or wrong*, *it is trial and error…”* (*Parent 12*)
	*“it’s a trial and error because*, *as you know*, *the diabetes for everyone is different and even if you have the basis of this physical activity affects this person or should affect someone that you might see completing the picture”* (*HCP 4*)
	*“…so I figured out what to do based on trial and error”* (*Adolescent 8*)
The work of parents: Parents actively support and advocate for their child’s diabetes management during PA.	*“sat through every single session … while she was swimming*, *I was….I spent my time researching*, *trying to find ways to help her”* (*Parent 8*)
	*“obviously my mum’s*, *like*, *she’s done a lot of it so she’s helped me*, *like*, *a lot … I don’t think I’m that knowledgeable … My mum is way*,*like … I definitely trust my mum more to know than I trust myself”* (*Adolescent 4*)
	*‘*cos *very often and*, *of course*, *it’s done because the parents worries and they want to … they want their kids to be healthy but we do see it*, *that that parents would do a lot instead of … er … their child and then they turn 18 or 19*, *they move … university or they start*,*umm … living on their own and you have to go back almost like re-diagnose because they know nothing or very little*. (*HCP 4*)
Learning from others: Advice and support from peers and the diabetes community helps families manage PA.	*“One of the places I learnt*, *probably the most*, *is Facebook … so*, *parent groups*, *umm … massively helpful*, *umm … in terms of realise….you know … understanding that you … you’re not alone…”* (*Parent 12*)
	*“what they need to find is other people that have similar outlooks*, *similar values*, *that*, *you know*, *that they can troubleshoot with someone that’s actually doing it..”* (*HCP 2*)
	*“there was a point where on Instagram*, *there are a load of diabetes*, *sort of*, *influencers. I found them quite helpful in a way that you could see another person living with your condition and they were talking about how terrible it was and how … what they do and I guess that sometimes helped to just be like*, *‘oh*, *so they’ve done this*, *should I try that?’…”* (*Adolescent 8*)
Support from others: Coaches, teachers, and peers can either ease or increase the burden of PA with T1D.	*“my coach was really good and he*, *like*, *he learnt all about it and*, *like*, *learnt what my numbers meant and looked*, *like*, *was always talking to me about it and stuff. He was*, *like*, *really invested in it”* (*Adolescent 4*)
	*“I think it’s trusting the people that you leave her with*, *as well*, *so I had a 100 percent faith in the coaches ‘*cos *they really were concerned and they really did watch and try to hel … learn theirselves and help.”* (*Parent 8*)
	*“there is resistance for youngsters with type 1 diabetes to be encouraged to do exercise because of fear of the caregivers of hypos*, *the fear of knowing what to do*, *umm … so*, *support for coaches*, *support for PE teachers about that*, *so that they can actually encourage the kids and know*, *like*, *know it’s okay for them to exercise and it’s gonna be fine* (*HCP 3*)*”*
Clinical support: Clinical advice varies, with some families receiving tailored guidance and others relying on general recommendations.	*“they’ll sometimes send out*, *er … a newsletter and it will just say*, *umm … you know*, *about help … exercise helping and to try and exercise but not actually giving any guidance in terms of how to manage your diabetes in relation to exercise*, *more just encouraging people to exercise..”* (*Parent 7*)
	*“when we spoke to*, *I … I can’t remember who … one of the consultants sent me … and we talked to the sports … person … who’s*, *like*,*…worked with people with diabetes*, *and I spoke to her to try and help with my swimming.”* (*Adolescent 4*)
	*“I don’t think it’s … er … adolescents friendly. I don’t think it’s adult friendly*. (*laughs*) *It looks like there is a lot of text*, *umm … and it’s very off-putting*, *umm … so I wouldn’t provide that to a young person…”* (*HCP 4*)

#### 3.2.1. Theme 1: How Much Mental Effort It Takes to Manage T1D When Being Physically Active

This theme explored the significant mental and emotional load associated with managing T1D in the context of PA. Four sub themes formed part of this overarching theme: (1) parental anxiety, (2) not wanting to feel different, (3) variability in response to activity or management, and (4) the complex decision‐making required for PA. (see Table 2 for a summary of these sub‐themes and illustrative quotes).

##### 3.2.1.1. Parental Anxiety

Participants described a worry from parents about hypoglycaemia, particularly when their children participated in unsupervised or group activities. This worry often made them extra watchful, closely checking blood glucose levels, making sure all supplies are packed, and often attending activities in person to be able to provide immediate support if needed. For some parents, this continued as their children got older and more independent, because they struggled to fully trust others, such as coaches or teachers, to handle emergencies properly.

Parental anxiety also influenced children’s participation, with some parents hesitant to allow independent activity due uncertainty about how others would respond in an emergency. HCPs noted that this anxiety could act as a barrier to promoting PA among young people with type 1 diabetes, particularly for younger children and those newly diagnosed.

##### 3.2.1.2. Not Wanting to Feel Different

Participants described a desire to avoid being singled out or perceived as different. This often intersected with issues of stigma and the visibility of diabetes technology (e.g., pumps, sensors). Many described strategies to conceal their devices, avoid drawing attention to their condition, or minimise disruptions during activities. The prospect of having to manage diabetes in front of peers, such as treating a hypo or adjusting equipment, was a source of embarrassment and could lead to reluctance to participate fully in PA.

The desire for normality was also reflected in the reluctance to join diabetes‐specific sports groups or to discuss diabetes openly with peers and coaches. Conversely both parents and young people described efforts to “just get on with it” and not allow diabetes to define their sporting experience.

##### 3.2.1.3. Variability: Unpredictability of Responses to Physical Activity

A dominant narrative across participants was the unpredictability and variability of blood glucose responses to PA. Both parents and young people described frustration at the lack of consistency, noting that the same management strategies for the same activity would often bring about different results on different days. As expected responses also varied depending on the type, intensity, and timing of activity. This variability contributed to a sense of cognitive overload, as families were required to constantly adapt, reflect, and problem‐solve in real time. Parents often expressed a desire for more tailored, activity‐specific guidance.

##### 3.2.1.4. Decision Making for Physical Activity and Negatives of Physical Activity With Type 1 Diabetes

Participants talked about having decide whether or how to take part in activity and having to constantly juggle things like adjusting insulin, planning meals and snacks, checking blood glucose levels. Making these decisions often felt like a burden and was frustrating, especially when managing diabetes got in the way of participating fully or performing well.

This decision making and complexities led to experiences of missing out on activities due to low or high blood glucose, feeling physically unwell during sport, or being forced to sit out while recovering from a hypo. Some young people reported pushing through symptoms to avoid letting down teammates, sometimes putting themselves at risk. Others described the “annoyance” of having to carry extra supplies, plan ahead, and having to accept that their experience of sport would always be more complicated than that of their peers.

#### 3.2.2. Theme 2: Practical and Organisational Challenges of Doing Physical Activity With Type 1 Diabetes

Managing T1Dduring PA involves complexities relating to both practical and logistical barriers that shape the daily experiences of young people and their families. These challenges are not limited to medical management but extend to the environments in which activity takes place, such as schools, clubs, and community settings, where understanding as well as access to support and resources can vary considerably. These demands often require ongoing problem‐solving, adaptability, and advocacy. As a result, the “work” of managing T1D around PA is substantial and can impact both the willingness and ability of young people to engage fully in sport and exercise. Two sub‐themes formed part of this overarching theme: (1) access (both in terms of activity and use of technology), (2) logistical barriers, and considerations needed to be active. (see Table 3 for a summary of these sub‐themes and illustrative quotes).

##### 3.2.2.1. Access

Access issues remain a fundamental barrier to participation in PA for young people with type 1 diabetes. These encompass the availability of diabetes management technology, the compatibility of devices with other equipment (such as smartphones), and the inclusivity of sports programmes and community initiatives. Families in deprived areas may lack the resources needed to support optimal diabetes management, and even when the diabetes technology is available (e.g., sensor), it is not always compatible with the family or young person’s existing technology (e.g., phone).

Alongside access to technology, participants described that some sports programmes or clubs are reluctant to include children with T1D due to perceived risks or a lack of confidence in managing acute situations, resulting in exclusion or limited opportunities for participation.

Participants described that support or initiatives to increase PA are often poorly tailored for young people with type 1 diabetes, with some programmes explicitly excluding those with health complications. Language barriers and lack of culturally appropriate support can further limit access for families from minority backgrounds.

##### 3.2.2.2. Logistical Barriers and Considerations Need

There are several, multi‐faceted logistical barriers and consideration relating to PA in type 1 diabetes. Adolescents and their parents described the challenges of carrying equipment such as snacks, drinks, testing kits, etc. as well as the added layers of complexity of managing T1D with PA relating to the unpredictability of activity schedules, the need to adjust insulin or carbohydrate intake based on the timing and intensity of exercise, and the lack of control over the structure of sessions. For example, young people may not know in advance what type of activity they will be doing in PE, making it difficult to plan insulin adjustments or carbohydrate intake. Other challenges reported included difficulties accessing supplies during activity, such as when bags are stored far from playing fields or when there is no suitable place to test blood glucose or treat a hypoglycaemic event.

Parents echoed the burden of preparation and the need for constant vigilance, noting that their children “constantly have to carry a bag” with all necessary supplies, and that even minor lapses in preparation can have significant consequences. The cumulative effect is a sense of extra effort and the need for planning and preparation simply to participate in activities that peers may take for granted and this affects the decision‐making process on whether to participate or not.

#### 3.2.3. Theme 3: Ways People Manage Their T1D When Doing Physical Activity

This theme looks at the different and changing ways young people and their families manage T1D during PA. Participants explained that there are no simple or set ways to manage it, instead, it involves constant learning, trying things out, and making changes. The way people manage often depends on their own experiences, how involved parents are, and the support they get from friends, the community and HCPs. The following sub‐themes illustrate the key elements of this adaptive process: (1) trial and error, (2) the work of parents, (3) learning from others, (4) support from others and (5) specific clinical support. (see Table 4 for a summary of these sub‐themes and illustrative quotes).

##### 3.2.3.1. Trial and Error

Adolescents, parents, and HCPs frequently described that managing T1D around PA is fundamentally a process of trial and error. While initial guidance, such as insulin reduction formulas or carbohydrate intake recommendations provides a starting point, individuals must repeatedly test, adjust, and refine their approach to find what works for them. This process is ongoing, as responses to PA can change with age, type of activity, and other life factors as described above in the subtheme *Variability: Unpredictability of Responses to Physical Activity*.

##### 3.2.3.2. The Work of Parents

Parents play a central role in supporting T1D management around PA. Many parents describe keeping meticulous diaries, researching strategies, and acting as advocates for their child in activity settings. As children grow older, parents often remain closely involved, providing reminders, reassurance, and practical support.

Parents and HCPs also describe the challenge of handing over management as children become more independent, emphasising the importance of gradually transferring knowledge and responsibility.

##### 3.2.3.3. Learning From Others

Peers and other families living with T1D are a highly valued source of practical knowledge and emotional support. Many parents, adolescents and HCPs describe the importance of seeking advice from friends, social media groups, diabetes camps, and community networks to supplement clinical guidance. These interactions provide real‐life examples, tips, and reassurance that others face similar challenges.

Young people also value opportunities to learn from others with type 1 diabetes, whether through formal camps or informal connections, and describe these experiences as normalising and confidence‐building.

##### 3.2.3.4. Support From Others

Support from schools, coaches, and peers was described as important but often variable. Some participants reported positive experiences with proactive and knowledgeable coaches or PE teachers. However, others highlighted gaps in advice and support. Adolescents and parents described both the value of supportive coaches and PE teachers and how when this is lacking it serves as a barrier to PA participation or places additional burdens on parents to support activity.

##### 3.2.3.5. Clinical Support

Clinical support refers to how diabetes teams help young people and their families manage T1D during PA. Adolescents and parents/carers described a range of different experiences, some received personalised, proactive support, while others said they were given little or no specific advice. HCPs also said the level of support can vary depending on funding, time, and staff interest.

Clinical support described by HCPs, parents and adolescents included dedicated sports clinics, structured education sessions, and tailored advice from clinicians with a special interest or expertise in exercise. For example, certain services offered regular appointments specifically to review patterns around sport, adjust insulin ratios for activity days, and provide practical strategies for different types of exercise:

Despite examples of good practice, several parents and young people reported that clinical support was often generic, limited, or absent. With little scenario‐based advice on how to adjust for different sports, intensities, or durations. Some were given written materials or brief verbal advice at diagnosis but found this insufficient as their needs evolved. Similarly, HCPs reported often lacking tailored resources for this population to help with support around PA, as well as describing lack in their own training to offer support.

## 4. Discussion

This qualitative study provides important insights into the complex and multifaceted experiences of adolescents with type 1 diabetes, their families, and HCPs in relation to PA. Our findings underscore the considerable cognitive, emotional, and practical burden associated with T1Dmanagement in the context of PA and highlight the need for more tailored and context‐specific support.

Our findings broadly align with the key themes identified in the 2016 qualitative evidence synthesis by Dash and colleagues [8], which reviewed 14 studies involving 270 participants including children and adolescents with type 1 diabetes, family members, teachers, and healthcare professionals. That review highlighted several core influences on PA participation, including blood glucose regulation, parental anxiety, individual motivation, social support, and the level of education and communication among stakeholders. The themes identified in the present study extend our understanding of several important factors including an up‐to‐date contextual understanding from multiple perspectives. Similar to Dash and colleagues we found that parental anxiety, often rooted in concerns around hypoglycaemia and perceived inadequacy of supervision by others, can limit independent participation and lead to heightened vigilance, sometimes at the expense of the adolescent’s autonomy. A notable addition to anxiety in our current study is the emphasis on the logistical and cognitive “work” required to manage diabetes around PA, which influenced decisions to participate in PA or not. Our study deepens understanding of how adolescents and parents learn through trial and error, emphasising the importance of interventions aimed to support self‐efficacy alongside providing the appropriate knowledge. Our data provide richer insights into the iterative, adaptive processes families use, showing how learning from successes and failures reinforces confidence and competence in managing PA.

Previous work by Vlcek and colleagues [22] explored strategies for maintaining an active lifestyle among adults and young people (over 16 years) with T1D and highlighted the importance of trial and error planning, and psychosocial support in managing PA with T1D as we have. By incorporating perspectives from parents and HCPs alongside young people themselves, our study also highlights the importance of the roles of parental advocacy, the variability in clinical support, and the influence of informal peer learning networks. These insights underscore the need for age‐specific, contextually tailored support strategies that address not only the technical aspects of diabetes management during PA but also the social and emotional realities of adolescence.

Peer support played an important role for many participants, who described learning from others through in‐person interactions and online communities. Digital platforms such as social media groups and virtual forums provided practical advice and emotional reassurance, helping adolescents feel less isolated in managing PA. Recent studies have shown that digital health Innovations such as virtual peer support, exergames, and tailored online communities can strengthen engagement and self‐efficacy. These approaches also help reduce feelings of isolation for adolescents with T1D [23, 24]. Future strategies for supporting PA in this population should consider integrating digital peer networks to broaden access and enhance support.

A prominent theme in our findings is the high degree of variability in glucose responses to PA, which led to frustration and a heavy reliance on trial and error. Importantly this was still described despite over half of adolescents in the sample using diabetes technology including CGM, pumps and closed loop systems. While trial and error has been recognised in the literature as a common part of diabetes self‐management, our findings suggest that for adolescents and families, this process can feel isolating and exhausting, particularly in the absence of clear, individualised guidance. The need for practical, scenario‐based education especially around different types of sport, intensities, and timings—emerged strongly across all participant groups.

Parental involvement was identified as both a key enabler and a potential barrier. Many parents described taking on a vigilant, hands‐on role to protect their child’s safety during activity, which sometimes led to over‐reliance and delayed transitions to independence. Conversely, the burden on parents, including the need to research, advocate, and plan, was significant. These findings support existing research on the central role of families in adolescent diabetes management [25] but add a new dimension by linking these responsibilities specifically to PA and its unpredictable demands.

Access to supportive environments including knowledgeable coaches, inclusive clubs, and understanding school staff—was varied. Some participants described highly supportive individuals who went out of their way to learn about diabetes and accommodate young people’s needs. Others encountered fear, exclusion, or a lack of confidence among facilitators, which sometimes resulted in young people being denied opportunities to participate. These findings reinforce concerns raised in previous UK‐based studies regarding the limited training and confidence among PA providers [26].

## 5. Strengths and Limitations

A key strength of this study is its multi‐stakeholder approach, capturing a broad range of perspectives across adolescents, parents, and HCPs from a variety of roles. This study aimed to capture perspectives from those most directly involved in family and clinical diabetes management and did not include the full range of external stakeholders such as PA providers, whose perspectives we have previously reported on [26]. The use of in‐depth interviews provided rich, contextual data, and the inclusion of younger adolescents fills a critical gap in the existing literature.

The emphasis on barriers in the first two themes may partly reflect the recruitment and data collection approach, which foregrounded barriers and challenges in the topic guide, and may have attracted participants who had experienced difficulties with PA management.

A key limitation of this study is that our sample was skewed towards families already engaging in PA, likely reflecting self‐selection among those with an active lifestyle or greater confidence around PA. Most parents who took part were mothers, echoing broader caregiving patterns in type 1 diabetes, where mothers typically assume primary responsibility for day‐to‐day management and support [27]. The sample was also predominantly White, limiting the transferability of findings to more ethnically diverse groups. Healthcare professionals interviewed were mainly dietitians and almost all were women, which reflects both the current NHS workforce composition and the fact that responsibility for PA support in paediatric diabetes care currently tends to fall to dietitians. These factors may restrict the applicability of our findings to other groups. Future research should aim to include a more diverse range of participants, particularly those from more disadvantaged and underserved groups.

### 5.1. Practical Implications

The findings point to a clear need for more individualised, flexible support strategies for adolescents with T1D engaging in PA. These should include scenario‐based education that reflects the realities of different sports, environments, and personal circumstances; structured support for parents to build confidence and gradually support the transition of responsibility to young people; training and practical resources for teachers, coaches, and community leaders to support safe and inclusive participation; and integration of peer‐led and community‐based models to complement clinical input. Future intervention development should prioritise co‐design approaches involving young people, families, and professionals, ensuring that support tools are relevant, accessible, and grounded in lived experience. Additionally, the implementation of standardised education for healthcare professionals in PA and diabetes management could help reduce current inconsistencies in care provision.

## 6. Conclusion

Adolescents with T1D face a complex interplay of cognitive, emotional, and practical challenges in managing PA. While families and peer networks provide critical support, current clinical resources are often insufficiently tailored to the realities of daily life. Addressing these gaps through individualized guidance, enhanced education, and peer support will be key to promoting sustainable PA engagement and improving health outcomes in this population.

## Ethics Statement

The study was conducted according to the ethical guidelines of the Declaration of Helsinki. Ethics approval was granted by the University of Exeter Research Ethics Committee prior to recruitment. Before taking part in interviews, participants were provided with an information sheet, and informed consent was obtained from all the participants.

## Conflicts of Interest

All the authors declare that they have no competing interest, financial or otherwise. The views expressed are those of the authors and not necessarily those of the NHS, the NIHR or the Department of Health and Social Care. RPD has received honoraria from Sanofi (Advisory Board on implementation of Teplizumab in clinical care).

## Author Contributions

Emma Joanne Cockcroft, Ross Clarke, Jenny Lloyd, Parth Narendran, Jane Rebecca Smith, Renuka Priyani Dias, Richard Pulsford, Louie Johnson and Robert Charles Andrews developed the study protocol. Emma Joanne Cockcroft conducted the interviews. Emma Joanne Cockcroft, Gina Gardener, Ross Clarke and Tallulah Ngamy conducted the analysis. Emma Joanne Cockcroft led writing of the manuscript. All authors reviewed and edited the manuscript for critical content and approved the final version. Emma Joanne Cockcroft was the principal investigator, had full access to all data in the study, and takes responsibility for the integrity of the data and the accuracy of the data analysis.

## Funding

This study was funded via an National Institute for Health and Care Research (NIHR) School for Primary Care Research Postdoctoral Fellowship (C010) award to the corresponding author (EC). RPD is supported by NIHR Award (Ref NIHR304587). This study was supported by the National Institute for Health and Care Research Exeter Biomedical Research Centre.

## Supporting Information

Additional supporting information can be found online in the Supporting Information section.

## Supporting information


**Supporting Information 1** Appendix 1: Consolidated criteria for reporting qualitative studies (COREQ) checklist.

**Supporting Information 2** Appendix 2: Example interview topic guide.

## Data Availability

The datasets generated and/or analysed during the current study are not publicly available but are available from the corresponding author on reasonable request.
