# Supplementary material for: Examining the Cognitive, Practical, and Emotional Demands of Managing Physical Activity in Adolescents With Type 1 Diabetes: A Qualitative Study With Adolescents, Parents, and Healthcare Professionals
Source: Pediatr Diabetes. 2025 Dec 19;2025:4578096. doi: 10.1155/pedi/4578096 (PMC12717440; doi:10.1155/pedi/4578096)
Supplement: Supplementary file 2 — Supporting Information 2 Appendix 2: Example interview topic guide. [file PEDI-2025-4578096-s002.docx]

| **Interviews with Adolescents**  **Topic Guide & Process**  **Version 0.1** |
| --- |

**Adolescent interview Topic guide**

Research objective: Explore the experiences of parents/carers of adolescents with Type diabetes around physical activity, as well as their views of intervention development. By physical active I mean things like walking to school, playing with friends or activities like Dance, surfing, orienteering, netball, football, or other activities when you’re moving your body.

**[Before turning recorder on]**

- Introduce myself
- Go over information sheet and provide details about the focus of the research
- State that the participant has previously provided Consent to participate in this study. Ask them to verbally consent to being involved with this interview.
- Explain process and that I will be making notes during the discussion as a reminder to myself to come back to
- Reiterate there’s no right or wrong answer. Interested to know about their experiences and views.
- Reassure participant that everything said during the interview will be anonymised
- Ensure that the participant understands that they do not have to answer any questions which they do not feel comfortable with. Explain that they can stop taking part at any time, without having to explain why, and can ask for a break at any time.
- Check whether the participant has any questions that they would like to ask.

**[Turn recorder on]**

**Theme #1: Experiences of physical activity**

1. **Could you tell me a bit about your what sort of activity you do ( or don’t)**
   1. How active are you?
   2. Where are you active? Are you active with friends and family or solo?
   3. Is being active important to you and your family?
      1. Enjoyment, managing diabetes, part of school, encouraged by family

**Theme #2: Support with T1D and physical activity**

1. Please can you describe support that you have you previously been given around managing your diabetes?
   1. Prompt: what does having this support mean for you? did you find it helpful? What was helpful / not helpful about it?
2. What about support specifically about managing your diabetes with physical activity?
   1. Prompt: what does having this support mean for you? did you find it helpful? What was helpful / not helpful about it?

**Theme #3: Barriers to physical activity**

1. What do you find Challenging about being physically active?

- *Prompts*:

| COM-B domain | Questions / prompts |
| --- | --- |
| Capability | Do you know how to be physically active whilst managing your diabetes? (Skills / knowledge)?  How do you decide when to do physical activity? |
| Opportunity | Do you have time to be active? Do you have the equipment / space to be active?  Do your school / home environment influence you physical activity levels?  Are you friends / family active? |
| Motivation | Is being physical active important to you?  Is it normal to be physically active?  How confident are you about being physically active?  Do you worry about being physically active?  If physical activity part of your normal lifestyle? |

1. Of the things that we’ve spoken about which are the most important challenges. Why is this?
2. What could be done to help you overcome this challenge?

**Theme #4: Enablers to physical activity**

1. What helps you to be physically active?

| COM-B domain | Questions / prompts |
| --- | --- |
| Capability | Do you know how to be physically active whilst managing your diabetes? (Skills / knowledge)?  How do you find following instructions about how to adjust insulin levels with physical activity?  How do you decide when to do physical activity?  Do you know how much physical activity you should be doing?  Do you know why you should be active? |
| Opportunity | Do you have time to be active? Do you have the equipment / space to be active?  Do your school / home environment influence you physical activity levels?  Are you friends / family active? |
| Motivation | Is being physical active important to you?  Is it normal to be physically active?  How confident are you about being physically active?  Do you worry about being physically active?  If physical activity part of your normal lifestyle?  What motivates you to be active? |

**Theme #5: Intervention ideas**

1. What things do you already do to get information about how to manage T1D?
   1. Websites, apps, social media

Prompts: why do you use this resource? Do you find it helpful? How could it be better?

1. Thinking about what challenges there are to being active what could we do to help support and encourage physical activity for young people with type 1 diabetes?
   1. Prompts – content (what should be included)
      1. Format (in person, app, website etc)
      2. If in person – where would it take place ( community, clinic etc)
      3. Who would deliver or if app / website – who would host it?

**WE’RE COMING TO THE END OF THE DISCUSISON NOW, AND I JUST HAVE A COUPLE OF CLOSING QUESTIONS**

- - - 1. Is there anything else you would like to say/add?
      2. Did you have any questions regarding today/the research?

*Please note that not all follow-up questions are listed in this topic guide. A number of additional questions may be asked based on the participant’s responses. Where possible, these will be open-ended questions.*
